# Bone cement-augmented vs. conventional pedicle screws for osteoporotic lumbar spondylolisthesis: a meta-analysis

**DOI:** 10.3389/fsurg.2025.1692448

**Published:** 2026-01-06

**Authors:** Guoyi Qin, Lihui Hu, Zhaoming Liang, Jinghuai Li, Xiaohang Bao, Shaohu Lin, Yicheng Wang, Yuanming Zhong

**Affiliations:** 1 Guangxi University of Chinese Medicine, Nanning, Guangxi, China; 2 The First Affiliated Hospital of Guangxi University of Chinese Medicine, Nanning, Guangxi, China

**Keywords:** lumbar spondylolisthesis, osteoporosis, bone cement, pedicle screw, meta-analysis

## Abstract

**Background:**

In patients with lumbar spondylolisthesis complicated by osteoporosis, conventional pedicle screw internal fixation often leads to complications such as screw loosening and fixation failure due to low bone mineral density. Bone cement-augmented pedicle screw technology has been widely used to enhance screw stability; however, its efficacy and safety in lumbar spondylolisthesis lack systematic evaluation.

**Objective:**

To systematically evaluate the clinical efficacy and safety of bone cement-augmented pedicle screws vs. conventional pedicle screws in the treatment of osteoporotic lumbar spondylolisthesis.

**Methods:**

Relevant literatures were retrieved from databases including PubMed, Cochrane Library, EMbase, Web of Science, CNKI, Wanfang, VIP, and SinoMed from their inception to July 2025. Retrospective cohort studies or randomized controlled trials were included. Two researchers independently screened literatures, extracted data, and assessed the risk of bias. Meta-analysis was performed using RevMan 5.4 software.

**Results:**

A total of 9 studies involving 850 patients were included. Meta-analysis results showed that compared with the conventional screw group, the bone cement-augmented screw group had significantly better outcomes in terms of the last follow-up Visual Analogue Scale (VAS) score (MD = −0.15, 95% CI: −0.22 to −0.08, *P* < 0.0001), Oswestry Disability Index (ODI) score (MD = −2.36, 95% CI: −3.98 to −0.75, *P* = 0.04), intervertebral space height (MD = 1.99, 95% CI: 0.73–3.25, *P* = 0.002), screw loosening rate (OR = 0.18, 95% CI: 0.09–0.38, *P* < 0.0001), and intervertebral fusion rate (OR = 3.98, 95% CI: 2.36–6.73, *P* < 0.0001). The operation time in the bone cement-augmented group was longer (MD = 32.13, 95% CI: 14.30–49.95, *P* = 0.0004); however, there were no significant differences in intraoperative blood loss, length of hospital stay, or complication rate between the two groups.

**Conclusion:**

Bone cement-augmented pedicle screws can significantly improve pain, functional status, intervertebral fusion rate, and screw stability in patients with osteoporotic lumbar spondylolisthesis. Although it prolongs the operation time, it does not increase the risk of intraoperative bleeding or complications, thus holding favorable clinical application value.

## 1 Introduction

Lumbar spondylolisthesis is a common spinal disorder characterized by the anterior displacement of one vertebral body relative to the adjacent inferior vertebra. It is one of the main causes of chronic low back pain and neurological dysfunction in middle-aged and elderly populations (1). Meanwhile, osteoporosis, a systemic skeletal disease characterized by reduced bone mass and deterioration of bone microarchitecture, shows a significant increase in prevalence with age, particularly among postmenopausal women (2, 3). The frequent coexistence of these two conditions poses substantial challenges to spinal surgical treatment. Although pedicle screw system internal fixation combined with intervertebral fusion (such as PLIF/TLIF) is a mature and effective surgical strategy for achieving spinal stability and neural decompression (4), its success rate is severely compromised under osteoporotic conditions.

The stability of pedicle screw fixation fundamentally relies on a robust bone-screw interface. Osteoporotic bones, due to low bone mineral density (BMD) and poor trabecular bone quality, significantly weaken the screw's holding power and pull-out resistance (5, 6). This biomechanical deficiency often translates into clinical complications, most notably screw loosening, displacement, and pseudarthrosis formation, which can lead to painful internal fixation failure, loss of correction, and even the need for revision surgery (7, 8). Reports indicate that the screw loosening rate in osteoporotic spines can be as high as 15.3%–38%, imposing a heavy burden on both patients and healthcare systems (9, 10).

To enhance the stability of pedicle screws in osteoporotic vertebrae, researchers have developed various techniques, including the use of expandable screws, bicortical fixation, hydroxyapatite-coated screws, and the addition of hook-rod auxiliary fixation (11, 12). Among these, augmentation with polymethyl methacrylate (PMMA) bone cement has emerged as one of the most popular and biomechanically effective solutions. This technique involves injecting bone cement into the vertebra through a hollow screw tract or a fenestrated screw, forming a reinforced cement-bone composite structure that significantly enhances the anchoring strength of the screw (13, 14). Biomechanical studies consistently demonstrate that, compared with conventional screws, bone cement-augmented pedicle screws can increase their axial pull-out force by 96%–200% (15, 16).

Despite promising biomechanical outcomes and increasing clinical application, there remains a lack of adequate comprehensive evaluation regarding the efficacy and safety comparison between cement-augmented pedicle screws (CAPS) and conventional pedicle screws (CPS) in the treatment of osteoporotic lumbar spondylolisthesis. While previous systematic reviews and meta-analyses, such as those by Rometsch et al. which focused on screw-related complications across the entire osteoporotic spine, and Cao et al. which examined osteoporotic vertebral fractures, have provided valuable insights, a dedicated synthesis of evidence specifically for osteoporotic lumbar spondylolisthesis—a condition with distinct biomechanical challenges related to instability and shear forces—is lacking. Our study aims to fill this specific gap by providing a focused comparison of CAPS vs. CPS in this patient population (17, 18). Lumbar spondylolisthesis, with its unique biomechanical requirements (involving instability and the need for robust fusion under shear forces) and clinical progression, deserves dedicated assessment. Furthermore, potential risks associated with bone cement augmentation techniques, such as cement leakage, embolism, and adjacent segment fractures, also warrant comprehensive evaluation of their safety in this specific patient population (19, 20).

Therefore, to provide high-level evidence for clinical decision-making, we conducted a systematic review and meta-analysis of comparative studies. This study aims to synthesize existing evidence to clarify whether cement-augmented pedicle screws can provide superior radiological and clinical outcomes compared with conventional pedicle screws in the surgical treatment of osteoporotic lumbar spondylolisthesis.

## 2 Materials and methods

### 2.1 Inclusion criteria for literature

#### 2.1.1 Study types

Retrospective cohort studies or randomized controlled trials, with the language limited to Chinese and English.

#### 2.1.2 Study subjects

Patients with spondylolisthesis who were eligible for posterior lumbar interbody fusion were included. Lumbar bone mineral density was measured by dual-energy x-ray absorptiometry, and those with a T-score <−2.5 SD were considered to have osteoporosis.

#### 2.1.3 Intervention measures

Intervention: The experimental group received treatment with cement-augmented pedicle screw fixation. The augmentation methods included cement augmentation through hollow screws and augmentation of conventional pedicle screws. The control group received treatment with conventional pedicle screw fixation. The bone cement material used in all cases was polymethyl methacrylate.

#### 2.1.4 Outcome measures

The outcome measures included operation time, length of hospital stay, postoperative Visual Analogue Scale (VAS) score, postoperative Japanese Orthopaedic Association (JOA) score, postoperative Oswestry Disability Index, intraoperative blood loss, postoperative intervertebral space height, postoperative drainage volume, screw loosening rate, complication rate, and postoperative intervertebral fusion rate.

### 2.2 Exclusion criteria

1) Literature without outcome measures; 2) summary literature such as abstracts and reviews; 3) literature with incorrect data and full text unavailable; 4) studies that are not clinical controlled trials; 5) literature without the required outcome measures; 6) summary literature such as abstracts and reviews.

### 2.3 Search strategy

Databases including PubMed, Cochrane Library, EMbase, Web of Science, CNKI, Wanfang, VIP, and SinoMed were searched for retrospective cohort studies or randomized controlled trials on the treatment of osteoporotic lumbar spondylolisthesis with cement-augmented pedicle screws. The search time span was from the inception of the databases to July 2025. Meanwhile, journal articles, dissertations, and conference papers were collected. The search method combined MeSH terms and free text.

Search terms included “Osteoporosi (#1)”, “osteopenia (#2)”, “lumbar olisthe (#3)”, “spondylolisthesis (#4)”, “lumbar slip (#5)”, “pedicle screws (#6)”, “pin (#7)”, “fixed (#8)”, “bone cement (#9)”, “strengthening (#10)”, “enhanced (#11)”. The search formula was: (#1 OR #2) AND (#3 OR #4 OR #5) AND (#6 OR #7 OR #8) AND (#9 OR #10 OR #11).

### 2.4 Evaluation methods

#### 2.4.1 Literature screening and data extraction

Two researchers independently conducted literature retrieval and screening. After duplicate checking using NoteExpress software, abstracts of literatures meeting the inclusion criteria were reviewed. After screening, the full texts of the remaining literatures were read for data extraction, followed by cross-validation by the two researchers. In case of disagreements, consultation and discussion with a third researcher were conducted. The extracted data mainly included: 1) first author and publication year; 2) inclusion criteria, demographic information, and baseline indicators of the study subjects; 3) study type and experimental methods (including implementation of blinding and randomization process); 4) study interventions (including drug dosage and course of treatment) and related outcome measures.

#### 2.4.2 Methodological quality assessment of literatures

Two researchers first assessed the study risk using the Cochrane Risk of Bias Assessment Tool. After cross-validation, any disagreements were resolved through consultation and discussion with a third researcher.

### 2.5 Statistical methods

Data analysis was performed using RevMan 5.4 software. For continuous data, the standardized mean difference (SMD) was used; for dichotomous variables, the relative risk (RR) was used, with 95% confidence intervals (CI) reported for both. A fixed-effects model was selected for analysis when there was good homogeneity among studies (*P* ≥ 0.1, *I*^2^ ≤ 50%). When there was significant heterogeneity among studies (*P* < 0.1, *I*^2^ > 50%), literature analysis was first conducted. If heterogeneity remained high after analysis, a random-effects model was used. For single-study reports, descriptive statistical analysis was performed. The Mean Difference (MD) was selected for all continuous outcomes instead of the Standardized Mean Difference (SMD) because the outcomes (e.g., VAS, ODI, JOA) were measured using identical, well-established scales across all included studies, making the MD a more direct and clinically interpretable measure of effect.

## 3 Results

### 3.1 Literature screening

**Figure 1:**
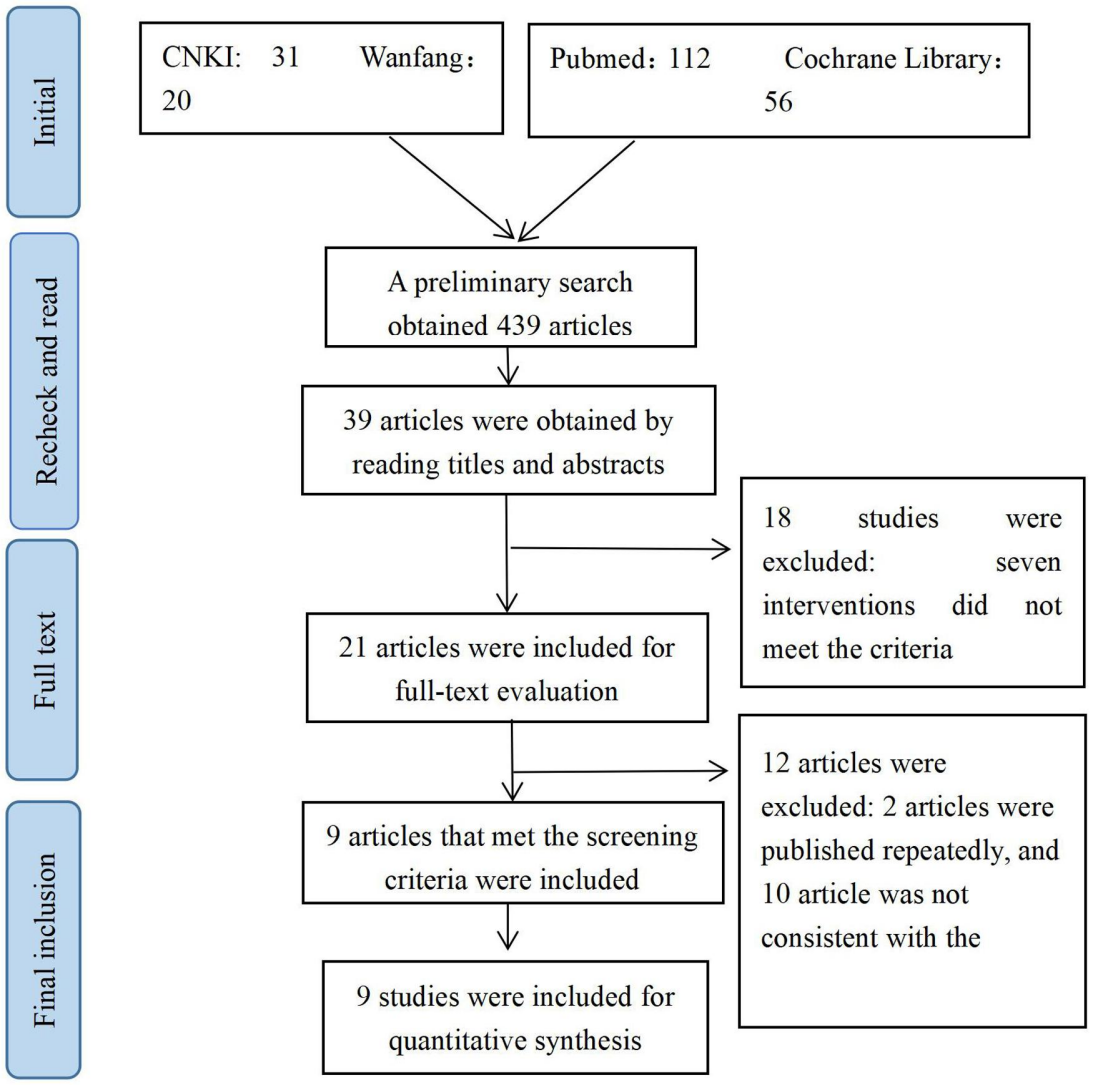
Document screening process.

**Figure 2:**
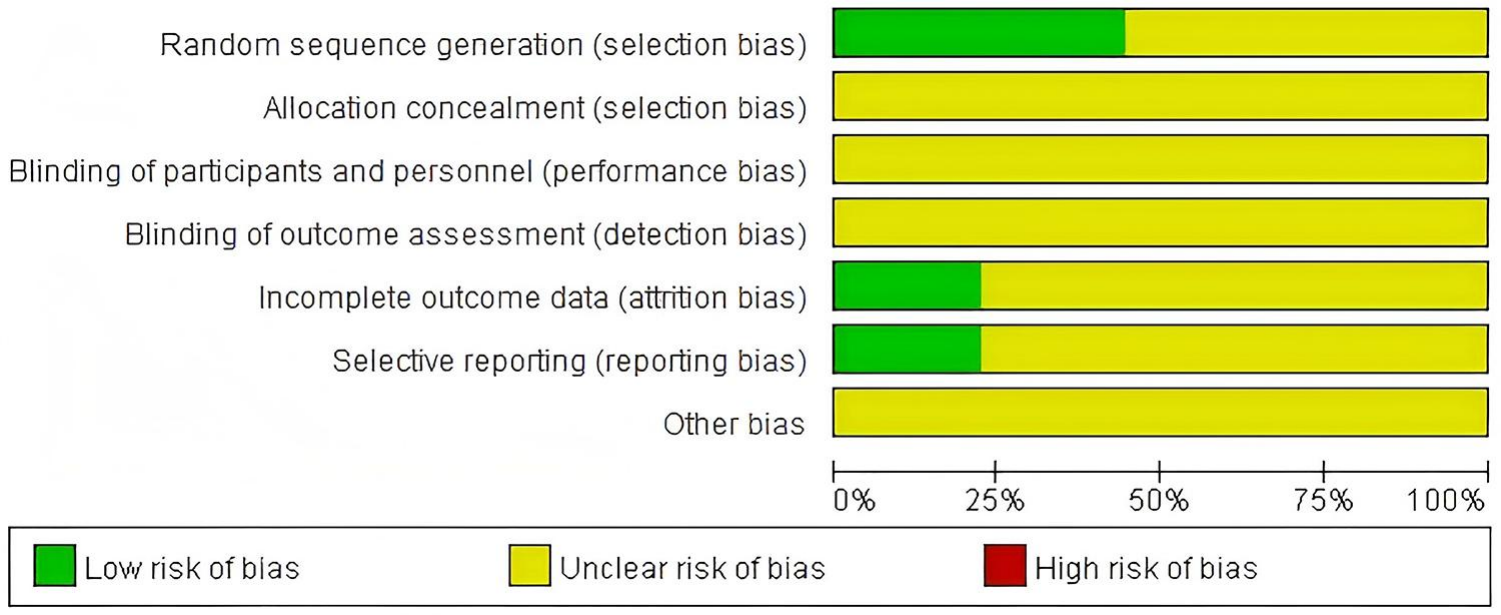
Proportion of items at risk of bias in the included literature.

Finally, 9 eligible literatures were included, with 3 being Chinese literatures and 6 being English literatures. All 9 included literatures were retrospective cohort studies, involving a total of 850 patients, with 424 patients in the bone cement-augmented screw group and 426 patients in the conventional screw group. Four studies mentioned varying degrees of bone cement leakage in the bone cement-augmented screw group, while others did not mention bone cement leakage. Nine studies reported that patients showed improvement after treatment in different sites. Four studies mentioned the occurrence of adverse reactions, including patients with superficial infections and cerebrospinal fluid leakage. No major adverse reactions such as nerve or spinal cord injury occurred in other studies. The screening process is shown in Figure 1. The basic characteristics of the included literatures are shown in Table 1. All 9 included literatures were none mentioned allocation concealment or blinding. The results of literature quality assessment are shown in Figure 2.

**Table 1:** Basic characteristics of the included literature.

Included literature	Sample size		Male/Female		Mean age/year		Interventions		Outcome measures
	T	C	T	C	T	C	T	C	
Chen et al. (21)	48	44	29/19	24/20	72.12 ± 4.39	72.06 ± 3.96	Bone cement-augmented pedicle screw	Conventional pedicle screw	①②③④⑤⑥⑨⑩
Cao et al. (22)	61	51	22/39	21/30	69.71 ± 11.65	64.42 ± 9.72	Bone cement-augmented pedicle screw	Conventional pedicle screw	②③⑧⑨
Zhou et al. (23)	21	22	5/16	7/15	65.42 ± 9.09	63.45 ± 7.07	Bone cement-augmented pedicle screw	Conventional pedicle screw	①②
Peng et al. (24)	33	26	7/26	8/18	64.67 ± 6.77	61.46 ± 6.26	Bone cement-augmented pedicle screw	Conventional pedicle screw	①②④⑤⑥⑧⑨
Wang et al. (25)	52	36	12/40	2/34	65.9 ± 9.38	67.3 ± 6.55	Bone cement-augmented fenestrated pedicle screw	Conventional pedicle screw	①②③④⑤⑦
Tian and Zhang (26)	41	41	10/31	8/33	55.23 ± 8.43	55.86 ± 7.24	Bone cement-augmented pedicle screw	Conventional pedicle screw	①②③④⑤⑥⑦⑧⑨⑩
Son et al. (27)	55	132	13/42	35/97	71.3 ± 7.0	67.2 ± 9.8	Bone cement-augmented pedicle screw	Solid pedicle screw	①②⑧⑩
Wang et al. (28)	85	46	3/82	3/43	69.22 ± 7.86	67.83 ± 6.05	Bone cement-augmented pedicle screw	Conventional pedicle screw	①②③⑦⑧⑨⑩
Mo et al. (29)	28	28	2/26	3/25	67.12 ± 1.31	66.04 ± 1.08	Bone cement-augmented pedicle screw	Conventional pedicle screw	①②④⑤⑥⑦⑧⑨

T represents the experimental group; C represents the control group; “-” indicates not specified; all baseline data are comparable; ① VAS score; ② ODI score; ③ JOA score; ④ intraoperative blood loss; ⑤ operation time; ⑥ hospital stay; ⑦ intervertebral height; ⑧ screw loosening rate; ⑨ intervertebral fusion rate; ⑩ complication rate.

The 9 included retrospective cohort studies had comparable baselines. All study subjects met the diagnostic criteria for lumbar spondylolisthesis, with a total sample size of 850 cases, including 424 cases in the experimental group and 426 cases in the control group see Table 1.

### 3.2 Meta-analysis

#### 3.2.1 Vas score

**Figure 3:**
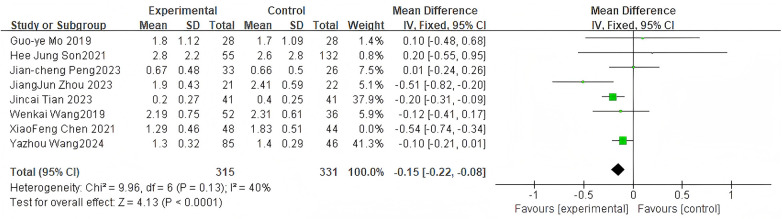
Forest plot of VAS scores between the bone cement-augmented screw group and the conventional screw group.

For the VAS score, 7 studies were included. The pooled analysis using a fixed-effects model showed a significant difference between groups (MD = −0.15, 95% CI: −0.22 to −0.08, *P* < 0.0001) as shown in Figure 3.

#### 3.2.2 ODI score

**Figure 4:**
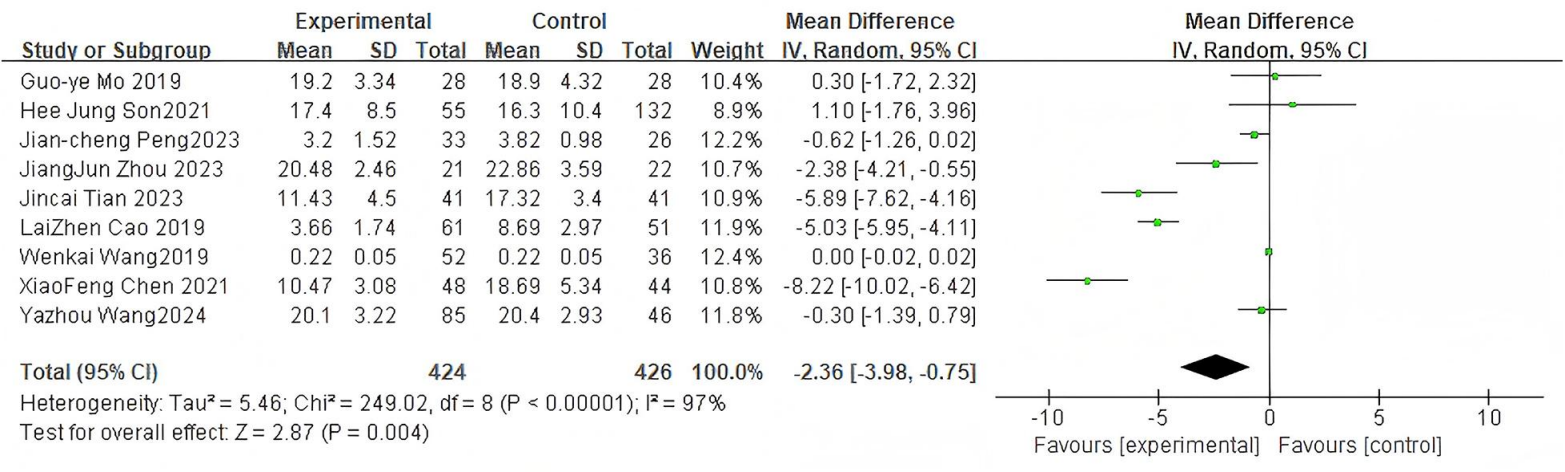
Forest plot of ODI scores between the bone cement-augmented screw group and the conventional screw group.

For the ODI score, 9 studies were included. The pooled analysis using a random-effects model showed a significant difference between groups (MD = −2.36, 95% CI: −3.98 to −0.75, *P* = 0.004) as shown in Figure 4. The substantial heterogeneity (*I*^2^ = 97%) may stem from clinical variations such as differences in cement augmentation protocols, surgical expertise, and patient characteristics across the included studies.

#### 3.2.3 JOA score

**Figure 5:**
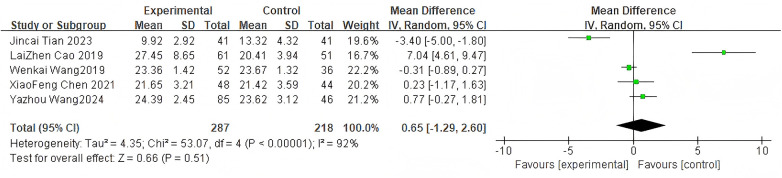
Forest plot of JOA scores between the bone cement-augmented screw group and the conventional screw group.

For the JOA score, 5 studies were included. The pooled analysis using a random-effects model showed no significant difference between groups (MD = 0.65, 95% CI: −1.29 to 2.60, *P* = 0.51) as shown in Figure 5.

#### 3.2.4 Intraoperative blood loss

**Figure 6:**
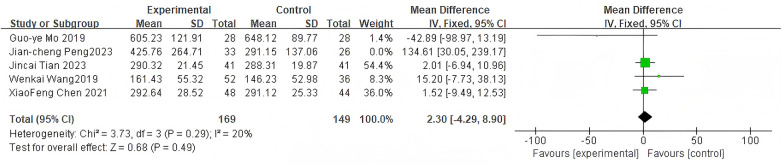
Forest plot of intraoperative blood loss between the bone cement-augmented screw group and the conventional screw group.

For intraoperative blood loss, 4 studies were included. The pooled analysis using a fixed-effects model showed no significant difference between groups (MD = 2.30, 95% CI: −4.29 to 8.90, *P* = 0.49) as shown in Figure 6.

#### 3.2.5 Operation time

**Figure 7:**
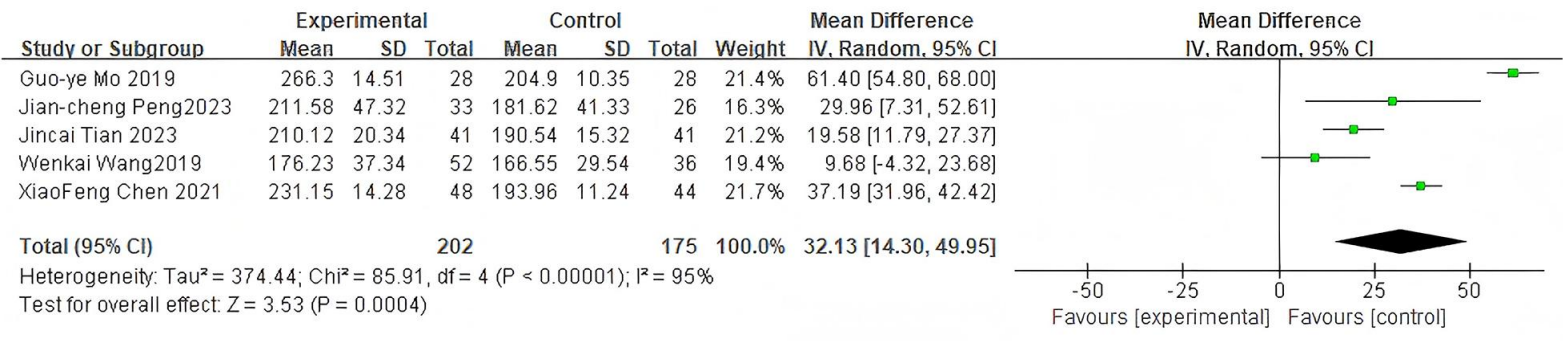
Forest plot of operation time between the bone cement-augmented screw group and the conventional screw group.

For operation time, 5 studies were included. The pooled analysis using a random-effects model showed a significant difference between groups (MD = 32.13, 95% CI: 14.30–49.95, *P* = 0.0004) as shown in Figure 7. The high heterogeneity (*I*^2^ = 97%) is likely due to methodological and clinical differences, including variations in the complexity of the cement augmentation technique itself, surgical team efficiency, and the learning curve associated with the procedure.

#### 3.2.6 Hospital stay

**Figure 8:**
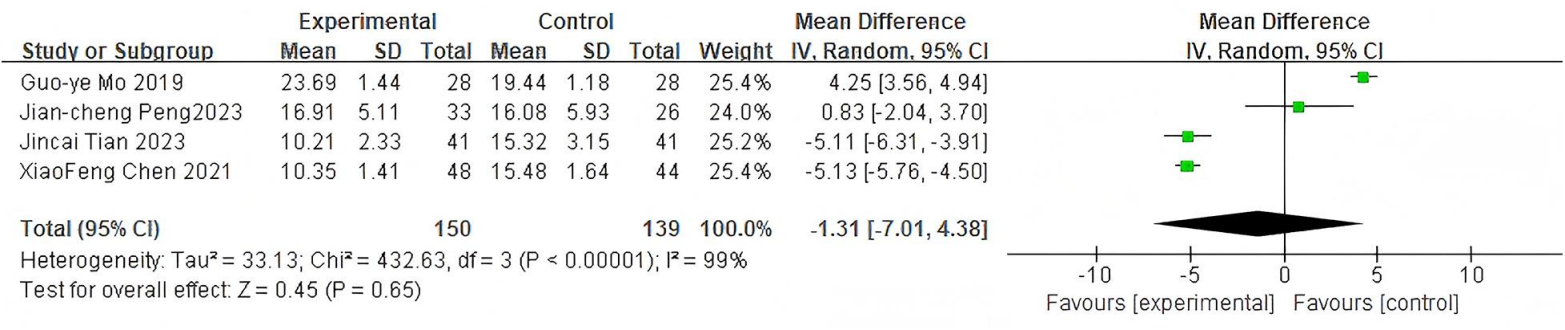
Forest plot of hospital stay between the bone cement-augmented screw group and the conventional screw group.

For hospital stay, 4 studies were included. The pooled analysis using a random-effects model showed no significant difference between groups (MD = −1.31, 95% CI: −7.01 to 4.38, *P* = 0.65) as shown in Figure 8.

#### 3.2.7 Intervertebral height

**Figure 9:**
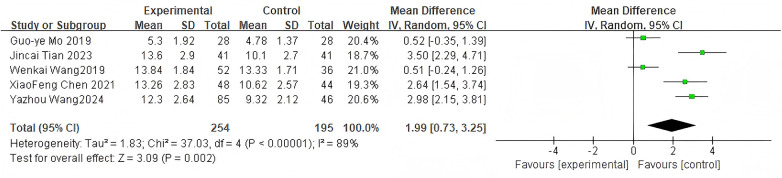
Forest plot of intervertebral height between the bone cement-augmented screw group and the conventional screw group.

For intervertebral height, 5 studies were included. The pooled analysis using a random-effects model showed a significant difference between groups (MD = 1.99, 95% CI: 0.73–3.25, *P* = 0.002) as shown in Figure 9.

#### 3.2.8 Pedicle screw loosening rate

**Figure 10:**
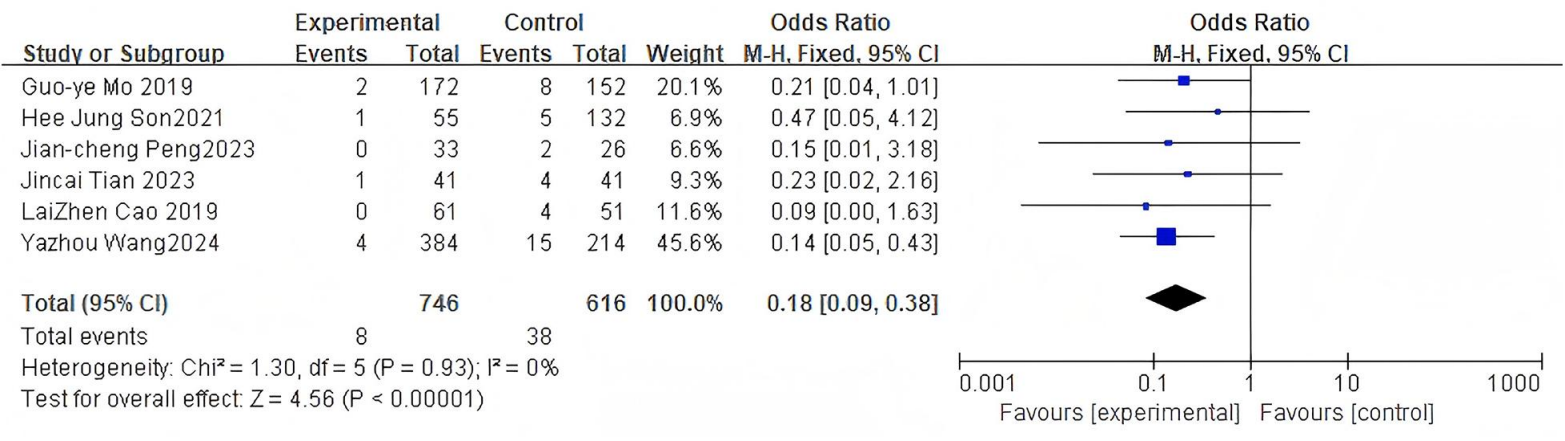
Forest plot of screw loosening rate between the bone cement-augmented screw group and the conventional screw group.

For the pedicle screw loosening rate, 6 studies were included. The pooled analysis using a fixed-effects model showed a significant difference between groups (OR = 0.18, 95% CI: 0.09–0.38, *P* < 0.0001) as shown in Figure 10.

#### 3.2.9 Intervertebral fusion rate

**Figure 11:**
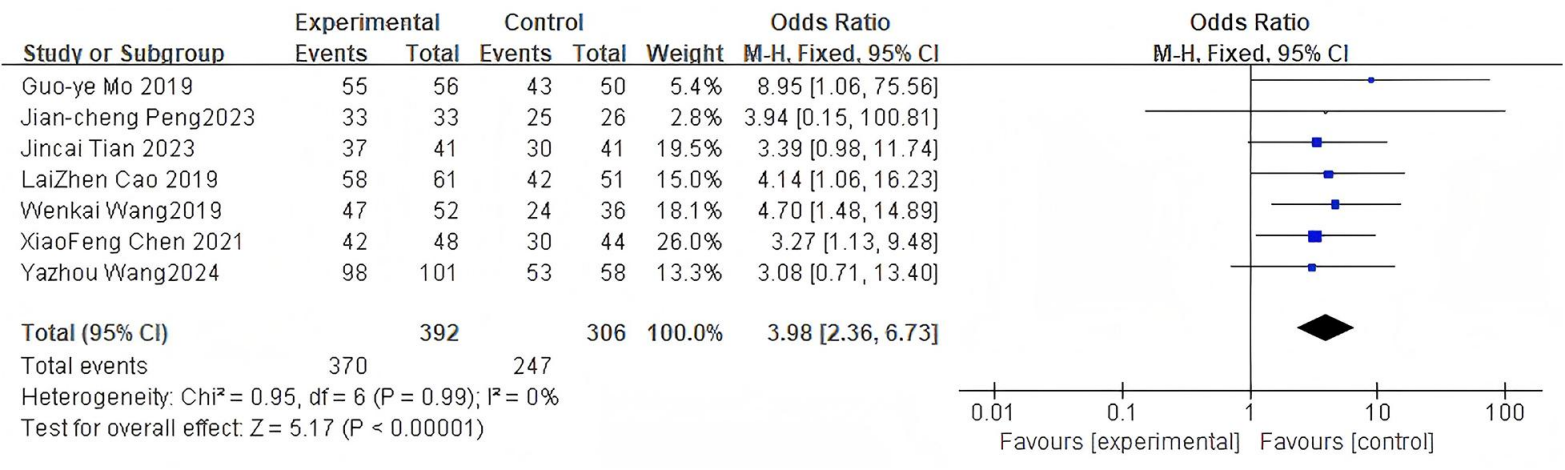
Forest plot of intervertebral fusion rate between the bone cement-augmented screw group and the conventional screw group.

For the intervertebral fusion rate, 7 studies were included. The pooled analysis using a fixed-effects model showed a significant difference between groups (OR = 3.98, 95% CI: 2.36–6.73, *P* < 0.0001) as shown in Figure 11.

#### 3.2.10 Complication rate

**Figure 12:**
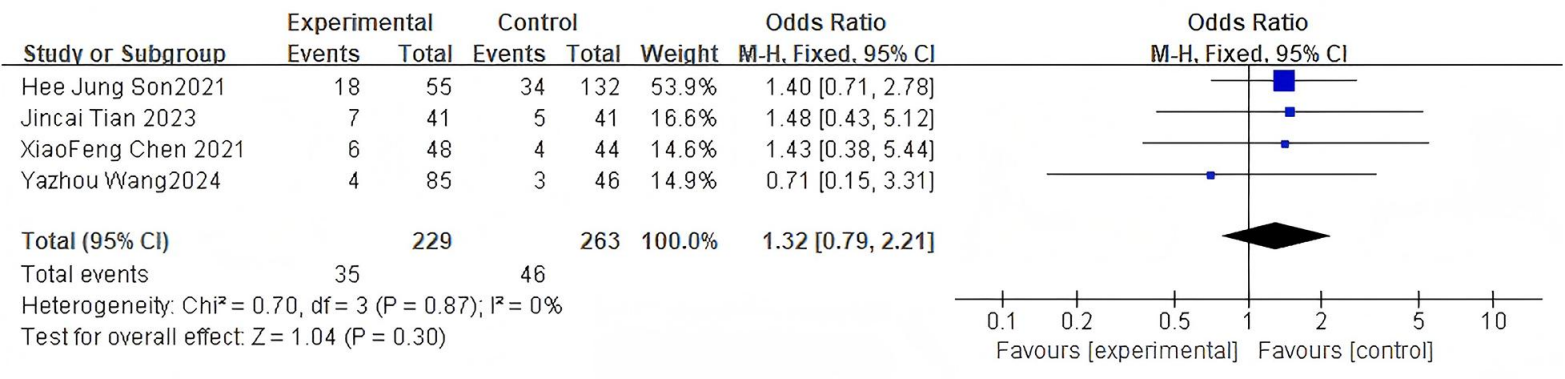
Forest plot of complication rate between the bone cement-augmented screw group and the conventional screw group.

For the overall complication rate, 4 studies were included. The pooled analysis using a fixed-effects model showed no significant difference between groups (OR = 1.32, 95% CI: 0.79–2.21, *P* = 0.30) as shown in Figure 12.

##### 3.2.10.1 Incidence of infection

**Figure 13:**
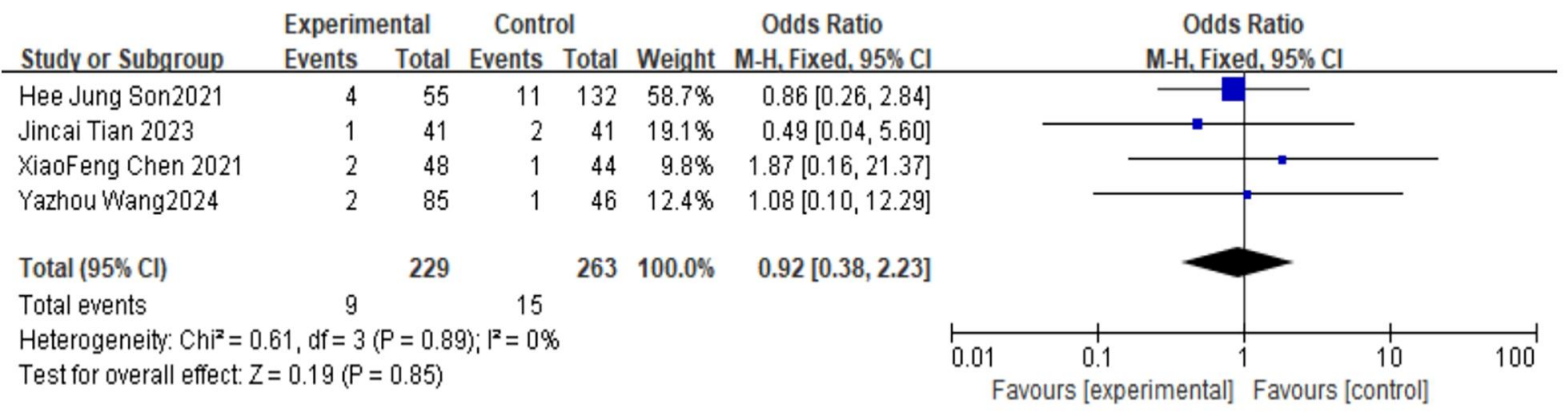
Forest plot of infection rate between bone cement-augmented screw group and conventional screw group.

For the incidence of infection, 4 studies were included. The pooled analysis using a fixed-effects model showed no significant difference between groups (OR = 0.92, 95% CI: 0.38–2.23, *P* = 0.85) as shown in Figure 13.

##### 3.2.10.2 Incidence of aggravated lower extremity neuralgia

**Figure 14:**
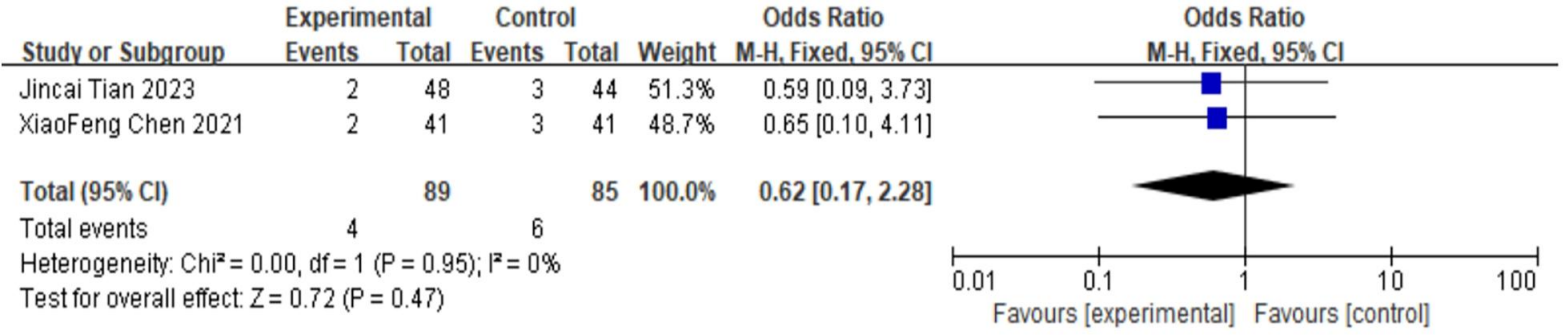
Forest plot of incidence of aggravated lower extremity neuralgia in the cement-augmented screw group versus the conventional screw group.

For the incidence of aggravated lower extremity neuralgia, 2 studies were included. The pooled analysis using a fixed-effects model showed no significant difference between groups (OR = 0.62, 95% CI: 0.17–2.28, *P* = 0.47) as shown in Figure 14.

### 3.3 Sensitivity analysis

For the operation time, after sequentially excluding each study and re-conducting the pooled analysis, no significant change in heterogeneity was observed, and the comparison results between the two groups remained consistent with the previous findings, indicating that the Meta-analysis results are relatively reliable. The high heterogeneity among studies may be attributed to differences in surgical segments, surgical difficulty, and the technical proficiency of surgeons across various studies.

For the hospital stay, sequential exclusion of each study followed by re-pooled analysis showed no significant alteration in heterogeneity, with the between-group comparison results consistent with the original findings, suggesting the reliability of the Meta-analysis results. The substantial heterogeneity among studies is likely due to variations in postoperative intervention measures and differences in patients' economic status across different studies.

Regarding the VAS score, sensitivity analysis indicated that one study might be a source of heterogeneity. After excluding this study, the re-conducted pooled analysis revealed a significant reduction in heterogeneity (by 30%). Using a fixed-effects model for analysis, the results showed a statistically significant difference in VAS scores between the two groups [MD = 2.30, 95% CI (−4.29, 8.90), *P* = 0.49], indicating that the Meta-analysis results are relatively reliable.

For the Oswestry Disability Index, JOA score, fusion rate, and intervertebral height, sequential exclusion of each study and re-pooled analysis demonstrated no significant changes in heterogeneity, with consistent between-group comparison results, suggesting the reliability of the Meta-analysis results. The heterogeneity among studies may be caused by differences in the duration of postoperative follow-up across various studies.

For intraoperative blood loss, sensitivity analysis suggested that one study might be a source of heterogeneity. After excluding this study, the re-conducted pooled analysis showed a significant reduction in heterogeneity (by 39%). Using a fixed-effects model, the results indicated that there remained no statistically significant difference in intraoperative blood loss between the two groups [MD = 2.30, 95% CI (−4.29, 8.90), *P* = 0.49], suggesting the reliability of the Meta-analysis results. The heterogeneity may be attributed to differences in surgical difficulty among the studies.

## 4 Discussion

This study conducted a Meta-analysis of 9 retrospective cohort studies, aiming to systematically evaluate the efficacy and safety of cement-augmented pedicle screws (CAPS) vs. conventional pedicle screws (CPS) in patients with osteoporotic lumbar spondylolisthesis. The inherent selection bias and confounding bias of retrospective studies cannot be completely avoided, which may affect the strength of evidence for the results. Therefore, the conclusions of this paper should be regarded as preliminary confirmation based on the best available but non-optimal evidence, and it is crucial to verify them through well-designed multicenter randomized controlled trials (RCTs) in the future. The comprehensive analysis results showed that, compared with the traditional technique, the CAPS technique can significantly improve patients' postoperative pain and functional status, better maintain intervertebral height, substantially reduce the risk of screw loosening, and increase the intervertebral fusion rate. Although the operation time in the CAPS group was longer, there were no significant differences between the CAPS group and the CPS group in terms of intraoperative blood loss, hospital stay, and overall complication rate. These findings provide evidence-based medical support for the application of the CAPS technique in the treatment of lumbar spondylolisthesis.

This study found that the screw loosening rate in the CAPS group was lower than that in the CPS group, and the intervertebral fusion rate in the CAPS group was higher than that in the CPS group. This result is highly consistent with the findings of Han et al. (30) and Mu et al. (31) in a broader range of thoracolumbar degenerative diseases, confirming the core advantage of bone cement augmentation in overcoming insufficient screw holding power caused by osteoporosis. The underlying mechanism lies in that the injected PMMA bone cement forms a solid “cement-bone” composite structure around the screw track, which greatly increases the contact area between the screw and the bone as well as the microscopic mechanical interlocking (32), thereby providing axial pull-out force and anti-rotational stability far exceeding those of conventional screws (33, 34). In the treatment of lumbar spondylolisthesis, firm internal fixation is a prerequisite for resisting shear stress and promoting successful bone fusion (35, 36). The results of this study indicate that the CAPS technique reduces the risk of internal fixation failure caused by screw loosening by providing a more stable mechanical environment, creates favorable conditions for biological fusion, which is reflected in the higher radiological fusion rate.

In terms of clinical outcomes, patients in the CAPS group reported better VAS and ODI scores at the last follow-up compared to the CPS group. Improvement in pain and function is a primary goal of spinal surgery (37), and we believe this improvement is likely directly related to the higher fusion rate and more stable internal fixation. A spinal segment that has achieved bony fusion can effectively eliminate pain caused by instability and allow patients to engage in functional rehabilitation exercises earlier and more actively, thereby achieving better long-term function (38, 39). It is worth noting that there was no statistically significant difference in JOA scores between the two groups, which may be related to the inherent characteristics of the JOA scoring scale, differences in assessment methods among included studies, or insufficient follow-up duration to reveal subtle functional differences in the scoring scale. In addition, this Meta-analysis observed high heterogeneity in indicators such as the Oswestry Disability Index (ODI), Japanese Orthopaedic Association (JOA) score, and operation time, which has multiple potential sources. At the technical level, the cement augmentation techniques adopted in different studies are not uniform, and may include various methods such as standard cannulated screw tract injection and fenestrated screws with side holes. These methods differ in cement distribution morphology and anchoring mechanism. Additionally, significant variations may exist among studies in terms of surgeons' operational experience, cement injection volume, viscosity, and timing of injection. These factors directly affect the results of functional scores, thereby introducing heterogeneity. Although we performed sensitivity analysis, these unmeasurable clinical and methodological differences remain the primary sources of high heterogeneity.

This study confirmed that the CAPS technique significantly prolongs the operation time (by an average of approximately 32 min). This is mainly attributed to the screw track preparation before cement injection, cement mixing, injection process, and the more careful intraoperative fluoroscopic monitoring necessary to prevent cement leakage (40). More importantly, this increase in time did not translate into an increase in other perioperative risks. There were no significant differences between the two groups in intraoperative blood loss, hospital stay, or overall complication rate. This indicates that when performed by experienced surgeons, the CAPS technique, although more cumbersome in steps, has controllable safety.Meanwhile, attention should be paid to specific complications directly associated with cement augmentation techniques, which reminds us of the potential risks of this technology. Cement leakage is one of the most concerned complications of the CAPS technique, and its consequences may include nerve compression or pulmonary embolism (41, 42). Furthermore, the physical and chemical properties of bone cement have a significant impact on surgical safety. In clinical settings, factors such as contamination of bone cement by physiological fluids during polymerization, or deviation of the mixing ratio of its monomer and powder from the recommended standards, may adversely affect the cement's polymerization process, final mechanical strength, and fatigue life. This further potentially impairs the long-term fixation effect of screws and increases the risk of complications. Recent studies have provided strong evidence for this, indicating that physiological fluid contamination significantly reduces the selected mechanical properties of acrylic bone cement, while component ratio imbalance similarly exerts a significant impact on the mechanical properties of medium-viscosity bone cement (43). These findings emphasize the importance of strictly adhering to bone cement usage specifications and optimizing operational procedures to ensure the safety of the CAPS technique.

We analyzed “cement augmentation techniques” as a whole. However, this technology is diverse in clinical practice. In addition to the aforementioned differences in screw design (e.g., fenestrated screws), the inherent properties of bone cement itself are also crucial. For instance, PMMA (Polymethylmethacrylate) bone cements with different viscosities vary in leakage risk. Furthermore, the addition of bioactive additives (such as hydroxyapatite, calcium sulfate, or strontium salts) to PMMA improves the biocompatibility of bone cement, promotes osseointegration, and can reduce the risk of long-term complications associated with traditional PMMA. Evidence suggests that different additives alter the mechanical properties of bone cement (44). These differences in technical details may potentially affect the long-term stability of screws and clinical outcomes.

Additionally, the included studies generally have a relatively short follow-up period. Although mid-term results show that CAPS has significant advantages in screw stability and fusion rate, its long-term durability and the risk of potential delayed complications remain unclear. For example, rigid internal fixation may alter the biomechanical distribution of the spine, increase stress on adjacent segments, accelerate degeneration, or raise the risk of adjacent vertebral fractures. Meanwhile, changes in the cement-bone interface after several years or even decades, as well as rare issues such as delayed infections, all require longer-term follow-up observation. Therefore, the current results mainly reflect the short-to-medium-term efficacy and safety of CAPS. Prospective studies with long-term follow-up are urgently needed to comprehensively evaluate the durability of the technology's outcomes and the actual risk of delayed complications.

Despite the valuable insights provided by this study, there are still limitations. Variations exist among the studies in terms of bone cement injection volume, viscosity, injection techniques, and follow-up duration, which may contribute to the high heterogeneity observed in certain indicators (such as ODI and JOA scores).Furthermore, the protocol for this systematic review was not prospectively registered, which constitutes a limitation to the reported methodology. Due to the limited availability of raw data, we were unable to conduct more in-depth subgroup analyses on different bone cement augmentation techniques. Future studies should focus on conducting multicenter, large-sample RCTs to provide higher-level evidence. Additionally, long-term follow-up is crucial for observing the incidence of long-term complications and adjacent segment diseases, which is also of great significance for the application of this technique.

## 5 Conclusion

Based on the current body of retrospective evidence, the use of bone cement-augmented pedicle screws in patients with osteoporotic lumbar spondylolisthesis is associated with superior mid-term radiographic outcomes (higher fusion rate, lower screw loosening) and improved patient-reported pain and function compared to conventional screws, without a significant increase in overall perioperative complications, albeit at the cost of longer operation time. However, given the inherent limitations of the included observational studies and the heterogeneity observed for some outcomes, these findings should be interpreted with caution. Future large-scale, randomized controlled trials with long-term follow-up are warranted to confirm these results and establish the long-term safety profile of the technique (45).

## Data Availability

The original contributions presented in the study are included in the article/Supplementary Material, further inquiries can be directed to the corresponding author.
